# P-392. Use of Oral Tobramycin to Prevent Surgical Site Infections in the Setting of an Oral Neomycin Shortage for Patients Undergoing Colorectal Procedures: A Retrospective Chart Review

**DOI:** 10.1093/ofid/ofae631.593

**Published:** 2025-01-29

**Authors:** Skyler Radziszewski, Melanie Altar, Nicole M Ondrush, Gopi Patel, Victoria Adams-Sommer, Eleanor D Sadler, Andrew Santeusanio, Emily Walits, Polina Lerner

**Affiliations:** Community Medical Center , Asbury Park, New Jersey; Mount Sinai Hospital, New York, New York; Mount Sinai Hospital, New York, New York; Icahn School of Medicine at Mount Sinai, New York, NY; Mount Sinai Hospital, New York, New York; Mount Sinai Hospital, New York, New York; Mount Sinai Hospital, New York, New York; Mount Sinai Hospital, New York, New York; Mount Sinai Hospital, New York, New York

## Abstract

**Background:**

Oral neomycin tablets have been the standard of care for gut decontamination to prevent surgical site infections (SSIs) in patients undergoing colorectal procedures. In August 2022, a shortage of oral neomycin tablets prompted the need for an alternative agent. Based on previously published data, intravenous tobramycin solution administered orally was selected at our institution. The objective was to compare rates of SSIs between oral neomycin and oral tobramycin in patients undergoing colorectal procedures.

**Methods:**

A single-center, retrospective cohort study was conducted in adult patients at The Mount Sinai Hospital who received preoperative oral antibiotics for gut decontamination prior to colorectal procedures. Patients who received oral neomycin between November 2021 and April 2022 were compared to those who received oral tobramycin between November 2022 and April 2023. Patients who did not receive a companion antibiotic (erythromycin or metronidazole) were excluded. The primary outcome was incidence of SSI within 30 days following colorectal procedure. Secondary outcomes included return to operating room (OR), re-admission, and mortality within 30 days of procedure, post-operative length of stay, and incidence of *C. difficile* infection.

**Results:**

A total of 119 patients were identified with oral gut decontamination regimens using neomycin (n = 63) and tobramycin (n = 56), who met study criteria. Baseline characteristics, including age, body mass index, and infection present at the time of surgery were similar between the two groups. The incidence of SSI was 3.2 per 100 procedures in the neomycin group and 3.6 per 100 procedures in the tobramycin group (p=1.00). There were no statistically significant differences in secondary outcomes including return to OR, readmission, incidence of *C. difficile* infection, or mortality within 30 days of the colorectal procedure. The median post-operative length of stay was 6 days in the neomycin group and 5 days in the tobramycin group (p=0.22).

**Conclusion:**

Intravenous tobramycin solution administered orally was not associated with a higher rate of SSIs than standard of care for decontamination prior to colorectal procedures. These findings will contribute to the limited literature on this topic in the United States.

**Disclosures:**

**All Authors**: No reported disclosures

